# What Factors Influence General Practice Specialist Trainees’ Engagement with their E-portfolio?

**DOI:** 10.15694/mep.2018.0000173.1

**Published:** 2018-08-17

**Authors:** Jonathan Rouse, Christopher Green

**Affiliations:** 1 Southend General Practice Specialist Training Programme; 2 University of Essex

**Keywords:** General Practice Training, E-portfolio, Medical Education

## Abstract

This article was migrated. The article was marked as recommended.

**Background:**E-portfolio is the primary strategy for the development and assessment of general practice trainees. Literature indicates that trainees often resist engaging fully, undermining its validity in assessing trainee competence and performance.

**Aim:**To identify and explore conditions influencing engagement with e-portfolio within a local training programme.

**Design:**A constructivist grounded theory design was used to conceptualise the factors that influence trainee engagement.

**Method:** Twelve semi-structured trainee interviews were audio-recorded, transcribed and coded. Aided by Nvivo Pro version 11, initial codes were developed, revised and raised to focused codes. Through constant comparison, diagramming and memo-writing, theoretical categories were generated.

**Results:**Data analysis conceptualised a theory of engagement incorporating three conceptual categories: “Conceptualising the e-portfolio”, “Developing and maintaining trust” in e-portfolio and its processes and “Deciding upon investment worth”. Decisions to invest personal resources in e-portfolio engagement depended upon trainees’ appraisal of its personal worth and value.

**Conclusions:**E-portfolio valuation was contingent upon trainees’ conceptualisation of its purpose, and the trustworthiness of the learning and assessment processes prescribed by its structure. This has implications for trainees, supervisors, and training programmes related to implementation and ownership of e-portfolio, and the credibility and transparency of its role in the assessment of professional performance.

## Introduction

The Royal College of General Practitioners (RCGP) ( RCGP, 2016a) defines e-portfolio as the modality for recording all workplace-based assessment (WPBA) and the “glue which binds the curriculum, learning and assessment”. E-portfolio forms one part of the “tripos” assessment allowing general practice (GP) trainees to achieve a certificate of completion of training, the other two components being national, standardised examinations ( RCGP, 2007). Some question the need for e-portfolio, citing the greater credibility of standardised examinations ( Lakasing, 2013), whereas, others suggest that objective measurement cannot adjudicate reliability ( Sadler, 1989; Rughani, 2008) and that portfolios are well placed to authentically and reliably assess performance at the highest level (“does”) of “Miller’s pyramid” ( Rethans *et al.*, 2002). WPBA can reliably assess trainee performance, however, reliability decreases as assessment numbers diminish ( Wilkinson *et al*., 2008). Similarly, reliability depends more upon regular sampling than a standardised test ( Van der Vleuten and Schuwirth, 2005). E-portfolio may therefore have greater reliability for assessing clinical performance, but only if engagement remains consistently high. Thus, understanding factors influencing engagement is important, both to assist individuals to excel and to quality assure assessment processes for external stakeholders.

Assessment is a key driver for learning ( Van der Vleuten and Schuwirth, 2005) and portfolios provide opportunities for learner feedback. However, as an assessment tool, portfolios reduce trainee ownership and self-confidence ( McMullan *et al*., 2003), and, transparent reflective log entries require sensitive handling, as judgements of those reflections may be viewed as judgements of character ( Tomlinson, 2015). Furthermore, trainees are more likely to present their best evidence, rather than displaying weaknesses when being assessed ( Norcini, 2003; Driessen *et al*., 2007), particularly considering the case of Dr Bawa-Garba ( Dyer and Cohen, 2018). This normative reflection rarely produces transformative learning ( Cotton, 2001).

Portfolio structure also influences its use in summative assessment. Less rigid structures have greater face and content validity ( Pitts, Cole and Thomas, 1999) and learners tend to favour portfolios which may be adapted to learning preferences ( Gray, 2008), however, summative assessment requires standardisation ( Snadden, 1999; Roberts, Newble and O’Rourke, 2002; Norcini, 2003). The rigidity of e-portfolio necessitated by its summative component may have paradoxically created barriers to engagement, negating formative benefits, and is worthy of further exploration.

Whilst early e-portfolio pilots were popular amongst self-selected cohorts, increasingly resistance is reported ( King, 2013), particularly regarding reflective writing ( Ruiz *et al.,* 2009). For trainees this issue is contentious given varying numbers of log entries required for each review period by different Local Education and Training Boards (LETBs) ( Osborne and Bal, 2013; Health Education East of England, 2015), despite the RCGP stating there is no minimum, just “..sufficient evidence of balanced curriculum and competence coverage” ( RCGP, 2016b).

Previous studies have assessed factors affecting e-portfolio use in medical undergraduates ( Ross, Maclachlan and Cleland, 2009; Belcher *et al.,* 2014) and across different specialties ( Kjaer, Maagaard and Wied, 2006; Tochel *et al*., 2009; Goodyear, Bindal and Wall, 2013) however good quality studies examining e-portfolio in GP training are lacking. There is also scant evidence available related to how peers, the formative and summative purposes of the portfolio, the structure of the portfolio and socio-political factors influence engagement. This study attempts to address this gap. Furthermore, this study aims to generate understanding of user perspectives and make recommendations to enhance the quality of educational support for trainees locally and nationally and provide feedback to the RCGP as to how e-portfolio might be developed to optimise engagement.

## Methods

This study employed a constructivist grounded theory design ( Charmaz, 2006) to identify and explore the factors influencing engagement with the RCGP e-portfolio within a local training programme. The study was conducted between October 2016 and September 2017 within a single general practice training programme. Trainees were invited to contribute to the design and subsequent reporting of the research, although uptake was limited.

In the interests of transparency several assumptions need to be made explicit. Firstly, the researcher subscribed to the educational value of portfolios. Secondly, the perception was that e-portfolio resistance was endemic amongst local trainees. These assumptions have important connotations regarding reflexivity. Thirdly, e-portfolio has arisen for its educational and regulatory value and is unlikely to be abandoned within medical education in the foreseeable future. Fourthly, engagement with e-portfolio was only considered quantitatively.

Consistent with “criterion-I sampling” (Palinkas *et al*., 2015), purposeful sampling was employed. Trainees with lower levels of portfolio engagement were sampled to provide insights into factors negatively influencing engagement. Low engagement was arbitrarily defined as less than half of locally expected numbers of WPBA and learning logs completed for stage of training, inactive personal development plans, stated concerns regarding engagement by the educational supervisor or unsatisfactory educational supervisor reviews. Trainees were contacted by email with an explanation of the purpose of the research and what participation involved. Subsequent emails and verbal invitations helped to increase participation.

Data were collected through twelve semi-structured interviews with twelve general practice trainees that were audiotaped and transcribed onto a secure computer. Focus groups were not deemed appropriate as there were risks that this method of data collection may suppress some trainees from disclosing useful data through emergent hierarchies ( Green, 2007). The initial interview schedule ( Box 1) was designed to explore identified gaps within a preceding literature review. All interviews were conducted by Jonathan Rouse. Consistent with grounded theory methods subsequent interviews were adapted based on initial analysis of earlier interviews. This provided richness and depth to the dataset.

**Box 1.** Initial Interview Schedule

**Previous experience of portfolio prior to GP training** **Support offered** - **Supervisor, training programme, deanery** **Attitudes and behaviours of peers** **Attitudes and behaviours of supervisors** - **Quantity over quality of learning logs** **Understanding the purpose of the e-portfolio** - **Sociopolitical factors** **Impact of the portfolio on personal development** **Place of portfolio within training** - **Compare with AKT and CSA, Summative assessment** **Factors affecting engagement** - **Time, rota, personal circumstances, IT** **Encouraging use/Improving motivation**

Data collection and analysis (aided by NVivo Pro v11 data management software) were performed simultaneously. Initial coding commenced following transcription of the first interview and continued throughout the interview process. Transcripts were coded initially, predominantly utilising gerunds to retain actions and decision-making processes, and, in-vivo codes to retain the trainees’ ‘voice’ throughout the analytical process. Initial coding remained open so as not to prematurely limit the analysis. The first six interviews produced over 1000 codes. Initial codes were compared and revised by amalgamating duplicates and modifying similar descriptors to capture ‘what was going on’ across data from different participants.

Focused coding was used to provide abstraction and conceptualisation ( Alemu *et al*., 2015). This involves selecting the initial codes which best represent the data’s core characteristics ( Sbaraini *et al*., 2011). Whilst the *a priori* literature review inevitably influenced this, merging certain codes and discarding others considered less relevant to the study’s aim provided a legitimate strategy for producing focused codes ( Saldana, 2016). In this study, focused codes incorporated the highly prevalent revised initial codes and described virtually the entire breadth of the data barring a few codes that appeared inconsequential to the study’s aims. Analytic memo writing provided comparative syntheses of coded data and shaped the analytic lines of enquiry. Memos supported the revision of initial codes, and, helped to develop focused codes and theoretical categories. Diagramming and mapping techniques were used to delineate interactions between categories.

By the eighth interview, repetitive themes persisted, and so interviews were paused to develop seven initial theoretical categories. A theoretical interview schedule was constructed ( Box 2) to uncover any disconfirming data, saturate categories and further examine how these were related. Data collected within four subsequent interviews did not produce any new categories but consolidated initial categorisation to three conceptual categories whilst also illuminating the links between categories.

**Box 2.** Theoretical Interview Schedule

**Assessing level of engagement** **Understanding of the primary purpose of the e-portfolio** **Who the portfolio is for** **Effect of trust upon engagement** - **How trust in the eportfolio and its systems is measured** - **How trust relates to understanding of the eportfolio’s purpose** **Relationship of trust and deciding upon investment worth** **Worth of the portfolio** - **Impact on engagement** - **Factors influencing perception of worth**

## Results/Analysis

The analysis illuminated the conditions and trainee decision-making processes that influenced engagement with the e-portfolio. Trainees made engagement decisions based upon their conceptualisation of the e-portfolio and the trust they placed in it as a learning tool. This determined the extent to which they were prepared to invest time, energy and resource into its development and completion. The category “Conceptualising the E-portfolio” pertained to socio-culturally influenced constructions of knowledge of how to use e-portfolio and personal understanding of its *raison d’etre,* which underpinned engagement strategies and their justification. “Developing and maintaining trust” in e-portfolio and its processes illustrated the influence of credibility of learning and assessment methods employed and variance between understanding of ownership and purpose with trainees’ lived experience. “Deciding upon investment worth” described how e-portfolio engagement was influenced by socio-culturally dependent cost-benefit decisions, whereby cost was measured in time and personal sacrifice, and benefit in relation to its perceived role and educational value. This ultimately influenced e-portfolio engagement.

### Deciding upon Investment Worth

*I think it’s time consuming. Time can be spent better elsewhere..ST3(4)*

E-portfolio engagement depended upon investment and worth, whereby investment comprised time, effort and personal sacrifice, and worth, the anticipated benefit. Trainees balanced the pay-off between spending time on reflection and the outcomes that this achieves:

*If you put the time in and you feel that you’ve really learnt something you don’t mind..ST3(3)*

Focused codes, such as “getting away with doing less” and “assessing personal gain” eluded to this cost-benefit decision-making. The focused code “juggling demands” highlighted that e-portfolio does not exist in isolation. Worth of investment is relative to other commitments, such as family, work and professional examinations:

*.. to find the time is quite difficult because.. you need to balance exam revision and also family life. ST3(7)*

Deadlines and summative assessment appeared to promote engagement, however, whilst this enhanced perceived worth of investment in relation to qualification, a focus on summative assessment may have been detrimental to reflective learning and development.

*..it was becoming more of a learning tool for me, but now it’s becoming more of..an exercise in completing and getting the numbers.. ST3(8)*

Time was identified as an important investment commodity needing to be found and spent. “Finding time” implied e-portfolio was of low priority and, for some, perceived worth was so low that even protected time would be considered wasteful.

*..do you think it would help if we had more time in a session. No, I think some people wonder that. ST3(4)*

Conversely, “spending time” suggested higher implicit value and greater personal investment. Participants expressed the view that time spent on e-portfolio should produce a return-on-investment. Reflective log entries required greater investment, as compared with WPBA and some participants implicated reflective learning logs as less reliable measures of their ability or performance, and less able to capture a trainee’s learning:

*I’d rather do more [WPBA], rather than learning logs.. I find it difficult to expand and write sentences about how I feel about that particular situation ST1(3)*

*..if it’s just clerking in a patient..then what is there to reflect on? ST1(2)*

Differences in implementation of reflective learning logs by supervisors and trainers further influenced how trainees perceived its worth and the extent to which they subsequently engaged:

*..if [others] can get away with 2 a month and we’re doing 8 a month..what’s that say about how that learning skill is viewed? ST3(3)*

### Developing and Maintaining Trust

*..there’s a lot that you can’t really see from just the portfolio. ST1(3)*

Developing trust in e-portfolio processes and trust in the ways in which these processes are implemented influenced the extent to which trainees committed to engaging with the portfolio. Trust was contingent upon trainees’ experiences - where there was clarity of expectation and consistent implementation, trust developed. This trust was fragile though and when trainees’ experiences contrasted with their expectation there was a risk that trust in e-portfolio processes would be lost and engagement diminished.

Inconsistent supervisor expectations and behaviours, knowledge of differing requirements in other regions and perceived lack of punitive measures for underperforming colleagues locally devalued engagement.

*..you do want people who don’t bother to have a bit more of a slap on the wrist..I don’t feel like I get a huge amount out of it..if I hadn’t of bothered it wouldn’t have made a huge difference. ST3(3)*

Differing standards promulgated subjectivity, reducing faith in e-portfolio as a summative assessment tool, and inculcated feelings of inequality, resulting in distrust and disengagement.

*.. there’s also unfair elements to it.. the expectations vary so massively.. and that definitely brings about a bit of disengagement. ST3(6)*

Trust was eroded through dissonance between the espoused value of the e-portfolio as a reflective, self-directed learning object based on adult learning principles and trainees’ experiences of it as a reductionist performance monitoring tool. Quantitatively measuring performance was often viewed as disempowering, which, through loss of a sense of ownership, undermined trust and engagement.

*..when you say there’s a set of numbers that you need to do then I think the intention changes from being purely reflective and learning something..ST1(2)*

*.. it’s part of our assessment, you need to still have.. the minimum number of learning logs, the minimum number of [WPBA], so I don’t really feel it’s like for me anymore.. ST3(2)*

Mutual trust was important, and trainees often expressed that espoused values of adult learning were lacking due to factors within e-portfolio and beyond. Platform inflexibility and seemingly irrelevant boxes reduced sense of ownership and stifled creativity.

*.. what puts me off writing entry logs.. is the fact that we have to chase our supervisors to read them.. you don’t want to annoy the person who’s your clinical supervisor.. ST1(2)*

*..I just draw mind maps and I just scan it on as my reading, but they still want me to write stuff in the bits like “why did you do this” .. ST3(2)*

Supervisor behaviours devaluing trainees’ learning and constant reminders regarding targets, without constructive qualitative feedback, undermined adult learning values and this perceived lack of mutual trust negatively influenced engagement.

*..there is lots of encouragement from supervisors telling us constantly do this, do that many learning logs, but that’s even more off-putting. ST3(3)*

Trust in supervisors was also influenced by the perceived ability of supervisors to assign relevant competences to log entries.

*I find it easy just to make sure it’s short and not too over-analysing stuff as people often miss a lot of stuff in there and just tick one or two boxes. ST3(5)*

For trainees to engage they needed to trust that e-portfolio accurately represented their capabilities. Trainees were more trusting of judgements made by those familiar with their daily practice but raised concerns regarding judgements made by external assessors.

*..they can use the logs..they can use the mini-CEXs and CBDs, but I still don’t think that it’s an accurate reflection. ST1(1)*

*.. I think the person that ultimately should have the say in who passes and who fails, based on your portfolio, should be your supervisor and not someone else who’s never met you.. ST3(6)*

Trainees’ trust in the methods used by e-portfolio to reflect their capabilities varied. There was a leap of faith that situations they selected to form the basis of reflections would be interpreted how intended - as instances of experiential and transformative learning that facilitated professional development. Some trainees were anxious that such reflections could be misused by assessors as evidence of poor practice and therefore poor performance.

*..you could come across as a bad trainee when you’re not a bad trainee because you’re reflecting on forced things that you’ve done wrong..whereas that’s not a true reflection of you, but you’re using it as a self-critical way of learning.. ST3(3)*

Trainees indicated that they were unsure of the confidentiality of records within the e-portfolio and who had access to their reflections. This directly impacted upon the kinds of situations trainees chose to include in their reflections, limiting potentially transformative learning opportunities.

*..there was a case gone to court about this..so, you don’t want to write, you know, too sort of important stuff ST3(5)*

### Conceptualising the E-portfolio

*It’s a place for us to be monitored, to see for supervisors and the deanery and, of course, our tutors to monitor what we’re doing. ST1(2)*

The ways that peers and supervisors conceptualised and implemented the e-portfolio were influential in constructing trainees’ knowledge and understanding. Where supervisors lacked awareness of the e-portfolio’s values and processes, trainees’ motivation to engage reduced.

*.. If the supervisor doesn’t know a huge amount about it, then [you’re] possibly less kind of motivated to do it..ST3(1)*

Peer interaction influenced conceptualisation and, despite a culture of negativity towards e-portfolio, shared knowledge and understanding appeared to enhance engagement.

*I don’t think that I’ve spoken to a single person who turns round and says this is an excellent thing..everyone hates it..ST3(2)*

*We try to sit and do it together with my other colleagues who are in GPST1 as well.. that’s been useful..ST1(3)*

Knowledge was conceptualised within procedural, systematic and contextual domains. Procedural knowledge, dependent upon induction and support from supervisors and the training programme, encompassed knowing how to use e-portfolio and how and when to write reflections. Overcoming engagement inertia was difficult without it.

*..I didn’t really know how to use it and then I didn’t know what I should be reflecting on, so just really basic things I didn’t know. ST2(1)*

Trainees’ expectations were manipulated by the cues they picked up from others.

*..one supervisor says, “yes I think you’re meeting that” and the other supervisor says “mmm, maybe, I see what you mean. ST3(4)*

Contextual knowledge comprised awareness of what trainees were doing locally in comparison with implementation processes reported in other regions. This sometimes led to a sense of injustice where trainees perceived differences in expectations between one context and another.

*..it’s unfair..whilst certain people in a different deanery will do a few logs we’re expected to do a lot more. ST3(6)*

The e-portfolio was conceptualised in terms of purpose and ownership too. E-portfolio was recognised as having developmental, pastoral and managerial purposes. Trainees developed an understanding of its primary purpose which influenced the investment worth attributed to engagement.

*..something had to be done..to show that the colleges are monitoring their trainees, and so it’s just created more paperwork to prove that they’re doing that..ST3(3)*

*..that’s why we have mini-CEX and CEPS and CBDs and these things, to really show that we’re not just working, but also learning..ST1(2)*

*..it’s also to kind of identify people who are struggling, or, um, possibly need a bit of extra support..ST3(1)*

Ownership was conceptualised at two levels. At one level, it concerned physical ownership of the platform and its relation to subscription fees and, on another level, it concerned actual content. An inflexible structure, quantitative measures of engagement and loss of control over access and disclosure tended to enhance the understanding of e-portfolio as being externally owned.

*I would be..happy just to write those 2 boxes and I could decide how much I wanted to write in each box.. ST3(2)*

### A Theory of GPST Engagement with the E-portfolio

**Figure 1.**
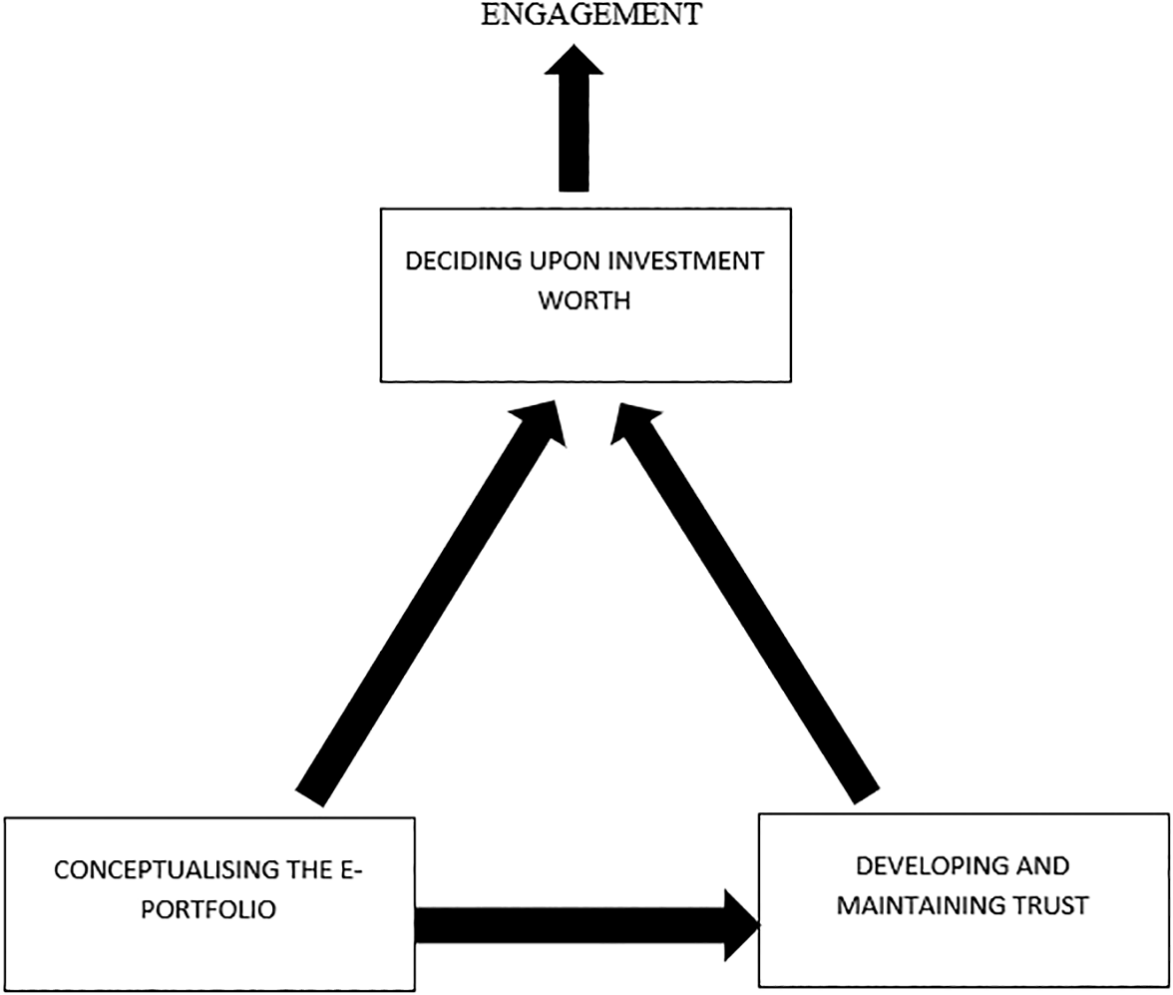
A Theory of GP Trainee Engagement with E-portfolio

This theory conceptualises e-portfolio engagement as ultimately being influenced by the perceived worth of investing in it. Several memos highlighted that value-based decisions were made by trainees at micro and macro levels regarding e-portfolio’s overall relevance and importance to their training and development. At a micro level, investment worth of specific components of e-portfolio were evaluated. At a macro level, investment was dependent upon how the portfolio was conceptualised, and the trust trainees placed within the portfolio and its implementation. Less energy was invested where mistrust of e-portfolio’s motives and processes existed, or if trainees felt that they were undervalued or treated unfairly.

*You go through the motions because you’re satisfying..this element that you can’t really control..if I thought my supervisor was the person that was going to be assessing me at the end that would be a far more useful assessment tool because then I’d give it more value. ST3(6)*

Understanding the purposes and beneficiaries of e-portfolio also informed evaluation of worth. Perception of e-portfolio as a performance management tool decreased investment relative to that as a personal development tool.

*..it’s..evidencing that we’ve trained you..if you’re actually that good, you can actually use it as a learning tool as well..ST3(5)*

Conceptualising the e-portfolio also informed development and maintenance of trust in this and its processes. This was undermined by knowledge of differing standards locally and in other regions, and, by e-portfolio’s perceived reductionism.

*..you can feel like you’ve created a good doctor..Look at all these doctors, they’re ticking off all these boxes..Does it actually mean that? My feeling is no, I don’t think it does.. ST3(4)*

Furthermore, understanding of purpose subsequently challenged by experience negatively influenced maintenance of trust in e-portfolio and its administration.

*It’s supposed to be a way for us to enhance our reflection, stimulate learning.. but then if it’s for us then why is it so didactic? Why is it so, enforced upon us..? ST3(6)*

## Discussion

### Summary

The findings of this study posit e-portfolio engagement as an investment decision, underpinned by the trust that trainees have within its learning and assessment methods and perceived inconsistencies in educational expectations. The ways in which trainees conceptualise the e-portfolio and its implementation influences trust and investment decisions. These provide the basis for evaluating congruence between expectations and experience and informing the cost-benefit decisions that mould engagement.

Initial preconceptions of the researchers were challenged, including that of engagement itself, highlighting qualitative dimensions, and producing reflection on how trainee engagement should be evaluated. The negative effects of quantitative reductionism that trainees characterise in this study appear to reduce personal investment and encourage superficial engagement in e-portfolio. This resonates with several extant psychological theories, which shall be explored below. It may be time to re-evaluate whether the RCGP e-portfolio fulfils its intended purposes for all stakeholders.

### Strengths and Limitations

This study addresses the gap within the literature pertaining to e-portfolio engagement since its widespread implementation in GP training. Significant overlap with prior work on portfolios and other extant psychological theories limits originality. However, the conceptual category “developing and maintaining trust”, which incorporates democratic issues, adds a further dimension to discourse on value and worth within medical education.

Although some interviews lacked full understanding of taken-for-granted meanings retaining participant voices throughout the analysis increased resonance, as indicated by participant feedback. Conceptual categories explained nearly all the data, however, revised coding may have narrowed data analysis earlier and limited potential depth.

Focusing on trainees with lower engagement levels means that these findings may have less relevance to those with higher levels of engagement. However, several practical recommendations were generated, and the abstraction of the conceptual categories appears to have reach beyond this specific context. Furthermore, e-portfolio informs trainee Annual Review of Competency Progression and the current review of this process ( Rimmer, 2017) may be informed by this research.

### Comparison with Existing Literature

Resonating with prior studies highlighting a positive correlation between perceived value and portfolio engagement ( Snadden and Thomas, 1998; Hrisos, Illing and Burford, 2008) this study identifies “deciding upon investment worth” as being the most influential factor for engagement amongst this study population. This may be explained by neoliberalist commodification of education and participant assumptions regarding learning and assessment as consumerist transactions ( Ingleby, 2015).

This study finds incongruent pedagogies in action that may adversely influence engagement. The contradictions inherent in reflective writing that do not allow for disclosure of experiences of suboptimal practice without risk of sanction is one example here previously observed amongst medical students ( Grant *et al*., 2006). Furthermore, lack of trust, loss of ownership and control appear to undermine autonomy, adversely affecting engagement and echoing findings amongst Dutch GP trainees ( Kjaer, Maagaard and Wied, 2006).

Amongst medical students, supervisor understanding of portfolio requirements was identified as being important for engagement ( Austin and Braidman, 2008), whilst perceived lack of supervisor clinical knowledge has been identified as a barrier to completing WPBA ( Hrisos, Illing and Burford, 2008). Similarly, this study suggests that supervisors whose knowledge and understanding of e-portfolio enables them to identify opportunities for WPBA and appropriately assign relevant professional competencies to learning logs positively influences engagement. Linking quantitative measures of engagement to supervisor ratings was apparent in this study, however, contrasting with earlier observations in which this was not directly linked to portfolio engagement ( Goodyear, Bindal and Wall, 2013) the findings indicate a negative impact when supervisors focus on quantity rather than quality. Supervisors who appear to value trainee learning and offer formative rather than “evaluative” feedback ( Dickinson, 1995), positively influence engagement. Sometimes supervisors may project the negative perception of the e-portfolio, with one trainee suggesting that the burden of e-portfolio upon supervisors is often transferred to trainees. This echoes previous findings ( Hrisos, Illing and Burford, 2008).

The findings of this study mirror those of others ( Grant *et al.,* 2006; Kjaer, Maagaard and Wied, 2006) in that time, competing demands and proximity of summative examinations all influence engagement. However, this study places these conditions within the context of an investment decision and therefore provides insight into value-based judgements that trainees make regarding e-portfolio engagement. Previous observations that engagement correlates with the perceived interest of individual cases ( Driessen *et al*., 2005) is also evident with many trainees suggesting that routine clinical contact provided minimal impetus for reflective log writing.

Trainees who value experiential learning and view e-portfolio primarily for learning and development suggest engagement would continue in the absence of summative assessment. Paradoxically, those perceiving it as a performance management tool, expressed opinion that if unassessed engagement would cease. However, summative assessment also appears demoralising, owing to a sense of autocracy. This, coupled with mistrust in how input is interpreted, produces superficial engagement. Process curricular models in medical education, whereby learning is assessment-driven ( Fish and Coles, 2005) should align with objectives and instructional activities to avoid under-recognition of teaching and learning ( Anderson, 2002). This combined with the ramifications of summative assessment for qualification ( Thompson *et al*., 2017) may explain this phenomenon.

“Deciding upon investment worth” is identified in this study as the main influence upon e-portfolio engagement. This has previously been recognised within the context of inter-professional learning when investment of personal resources appeared dependent upon “..perceived value of specific learning processes and outcomes” ( Green, 2013), and, in online learning when the “propensity to study” was influenced by its value and associated costs ( Scott, 2007). Braskamp (1986) described “Personal Investment Theory” amongst university students suggesting that “personal investment”, defined as behaviour and choice to engage in specific activities, was influenced by “personal meaning” constructed through pre-existing understanding and context ( Burton, 2012). This resonates with the socially constructed understanding of e-portfolio identified in this study.

The “Theory of Planned Behaviour” ( Ajzen, 1991) offers explanation of observable behaviours. The main determinants of an intended behaviour are thought to be “attitudes towards that behaviour”, “subjective norms” and “perceived behavioural control” (the ease or difficulty of executing the behaviour) ( Archer *et al*., 2008). The “Theory of Planned Behaviour” has been used to explain e-portfolio acceptance using a devolved model ( Ahmed and Ward, 2016) in which “attitudes and behaviour” related to perceived ease of use, usefulness and compatibility with learning preferences, “subjective norms” to superior and peer influences, and “perceived behavioural control” to facilitating conditions and self-efficacy. Many of these findings echo those of this theory of e-portfolio engagement.

### Implications for Practice

A summary of recommendations is shown below.

**Table 1.** Summary of Recommendations

	Hospital Clinical Supervisors	Educational Supervisors	Training Programme	LETB	RCGP
**Conceptualising the E-portfolio**	Provide e-portfolio training at faculty group meetings to develop a shared consensus on purpose and implementation		Increase time at induction and throughout the programme spent on e-portfolio Harness peer support by providing formal sessions and encouraging social networking		
**Developing and Maintaining Trust**	Highlight importance of positive attitudes towards e-portfolio.	Calibration and capacity building during trainers’ workshop		Uncover conditions that have led to differing regional expectations.	
**Deciding Upon Investment Worth**	Practice assigning professional competencies at faculty group meetings	Encourage constructive formative feedback rather than evaluative			Discussion regarding making the components of the e-portfolio more flexible

### Hospital Clinical Supervisors

Clinical supervisors should receive regular training on implementing e-portfolio effectively, assigning professional competencies to log entries, identifying when and how to undertake WPBA and encouragement to be pro-active in initiating WPBA for trainees. The importance of supervisors espousing the values of e-portfolio should be highlighted.

### Educational Supervisors

Issues regarding evaluative feedback and varying expectations should be fed-back to GP trainers at local trainers’ workshops. To avoid paradoxical disengagement, educators’ notes should be qualitative rather than quantitative. During workshops, activities could be undertaken that calibrate individual trainer’s standards, for example, analysing portions of anonymised e-portfolios.

### General Practice Specialty Training Programme

Shared understandings around implementation and expectations are needed amongst stakeholders. This may be achieved through consolidated induction and supplemented with written guidance and facilitated support sessions within half-day release programmes, helping those unable to attend. Peer support may be encouraged by providing formalised opportunities during protected half-day educational sessions for drawing on shared reflective practices that can be used to directly deliver on one element of e-portfolio and encouraging the use of social networking outside of these sessions to enhance knowledge and understanding.

### LETB

These findings suggest that differing e-portfolio expectations between LETBs negatively influence engagement. It is unclear why these discrepancies exist, and this should be discussed between LETBs to identify potential policy changes or whether greater transparency is needed.

### RCGP

The RCGP should discuss the (in)flexibility of e-portfolio such that potential changes, specifically to the learning log section of the platform, could be considered. Less restrictive reflective space with prompts, as opposed to mandatory fields, may enhance engagement.

### Implications for Future Research

This study finds that supervisor behaviours influence engagement. Understanding reasons for varying supervisor expectations may produce strategies for decreasing variance. Exploration of the conditions influencing supervisor expertise of e-portfolio and attitudes towards it may identify systematic and cultural barriers to be addressed. Identifying the factors which promote quantitative over qualitative supervisor feedback is important if the latter is to be encouraged.

Seemingly a large area of contention identified is the differing e-portfolio requirements between regions. Several trainees discovered this information informally and speculation abounds as to the reasons for it. In the interests of transparency and democracy a full realist evaluation of e-portfolio implementation and outcomes is suggested.

## Conclusion

The value attributed to the e-portfolio was contingent upon trainees’ conceptualisation of its purpose, and the trustworthiness of the learning and assessment processes prescribed by its structure. This has implications for trainees, supervisors, training programmes, LETBs and the RCGP. These implications concern implementation and ownership of the e-portfolio, and the credibility and transparency of its role in the assessment of professional performance.

## Take Home Messages

• Portfolios are described within the medical literature as having formative and summative purposes.
• Engagement is highly variable with limited evidence of why this happens.
• This study provides empirical evidence of barriers conceptualised as an investment decision.
• Investing personal resources depends upon individual conceptualisation of portfolios and the extent to which they trust the learning and assessment processes embedded within.

## Notes On Contributors

Dr Jonathan Rouse has been a general practitioner for the past 14 years in England. During this time, he has helped to educate medical students, local foundation doctors and latterly general practice trainees. For the past 5 years he has worked as a general practice training programme director.

Dr Christopher Green is programme lead for the MSc in Medical and Clinical Education (MaCE) in the School of Health and Social Care at Essex University. He sits on the Best Evidence in Medical Education (BEME) Board and is associate editor for the Journal of Interprofessional Care.
