# Supplementary material for: What Factors Influence General Practice Specialist Trainees’ Engagement with their E-portfolio?
Source: MedEdPublish (2016). 2018 Aug 17;7:173. [Version 1] doi: 10.15694/mep.2018.0000173.1 (PMC10701812; doi:10.15694/mep.2018.0000173.1)
Supplement: Peer review history

# Reviewer report: Reviewer response for version 1

**DOI:** 10.21956/mep.19642.r29271

**Published:** 2018-09-02

**Authors:** Ian Wilson

**Affiliations:** 1 International Medical University

This review has been migrated. The reviewer awarded 3 stars out of 5 I agree with Masters and Gibbs in their reviews. I would particularly like to emphasise the problems associated with the transfer of results in one region to the entirety of the program. this is problematic in this paper where it is evident that the manner of implementation varies between regions.As I work in Australia I had particular difficulty with the unexplained abbreviations. I presume the S in ST1 etc stands for Speciality. And I have no idea what LETB means.In summary an interesting article of limited impact due to its focus on one region.

Reviewer Expertise:

NA

No decision status is available

# Reviewer report: Reviewer response for version 1

**DOI:** 10.21956/mep.19642.r29269

**Published:** 2018-08-25

**Authors:** Ken Masters

**Affiliations:** 1 Sultan Qaboos University

This review has been migrated. The reviewer awarded 3 stars out of 5 Good detailed description of the Grounded Theory process applied.The major problem I have with the paper is trying to grasp the results, as the relationship between the quotations and the descriptions is really difficult to follow. They begin by being laid out with quotation and then description. • Firstly, it might be easier for the reader if this were reversed (as is fairly standard in most GT studies I have read). Otherwise, the quotation is read with almost no context, and then makes sense only when the description is read. In order to better understand the paper, I found myself skipping the quote, going down to the description, and then back up to the quote. And sometimes there are two quotes, and it is unclear how these relate to the descriptions. • Secondly, the order then appears to shift, as a description is given, followed by a colon and then an illustrating quotation. As an example, the sub-section headed “Conceptualising the E-portfolio” begins with quote followed by description, but then ends with a final quote, so the reader does not know if there is a description missing, or if that quote relates to the description above it. It might have helped if the authors had put in extra spaces, to group description and quote/s, so that these could guide the reader.• And sometimes, finding the connections between the descriptions and the quotations appeared very obscure. This may be because the authors are deeply familiar with the original material, and the connections are obvious to them. The connections, however, are not always obvious to the reader. For example, in this set, it is almost impossible to determine the relationship between description and quotation.---[Description]Trust in supervisors was also influenced by the perceived ability of supervisors to assign relevant competences to log entries.[Quotation]I find it easy just to make sure it’s short and not too over-analysing stuff as people often miss a lot of stuff in there and just tick one or two boxes. ST3(5)[Description]For trainees to engage they needed to trust that e-portfolio accurately represented their capabilities. Trainees were more trusting of judgements made by those familiar with their daily practice but raised concerns regarding judgements made by external assessors.[Quotation]…they can use the logs…they can use the mini-CEXs and CBDs, but I still don’t think that it’s an accurate reflection. ST1(1)----In cases like this, it might have been useful if the authors had edited the quotation with square brackets, making the designations of the pronouns clearer (e.g. they [the assessors/staff/students]).So, I found myself often having to gloss over much of the Results section, trying to form a general impression only. This meant, in turn, that I had difficulty evaluating the Discussion, because I could not reasonably form an opinion on whether or the Discussion was a true reflection of what was shown in the Results.I would strongly recommend that, in the revised version of their paper, the authors:• Clearly indicate the grouping of quotation and description.• Preferably give description before quotation.• Have their Results read by a person unfamiliar with the study and the original material, to ensure that the descriptions are accurately reflected in the quotations.Small correction:“eluded to this cost-benefit” should be “alluded to this cost-benefit”

Reviewer Expertise:

NA

No decision status is available

# Reviewer report: Reviewer response for version 1

**DOI:** 10.21956/mep.19642.r29270

**Published:** 2018-08-18

**Authors:** Antony Willman

**Affiliations:** 1 Bulford MTF, Defence Primary Healthcare

This review has been migrated. The reviewer awarded 4 stars out of 5 There is so much that is good about this paper. As a GP Trainer myself, I recognise a lot of what is reported. The study is well constructed and raises some excellent points.As a Trainer using the old structured trainers report, then transitioning to the e-Portfolio after a period not training, it is interesting to compare the formative vs summative role. This is brought out nicely. There is a certain amount of hoop jumping I think we all agree but this correlates with engagement.A semi-structured interview was I think the correct way to go as opposed to focus group - it is a very personal thing and even in small groups, this may bar GPSTs telling the whole story.It would be interesting to run a similar study on over-performing GPSTs or those who were in the top tranche of their MSRA to see if different themes emerge. Another group to possibly look at would be 1st or 2nd year First5 GPs. This might give some triangulation.How many entries are enough is a perennial question which does seem to vary. The variation would directly impact 'investment worth' especially as it is a question often asked of me as an ESR. If I don't know the exact number, what does that say? Again this is brought out nicely.Limiting it to one training programme may skew the results but somehow I doubt this based on the anecdotal experience of my own and my colleagues during discussions at Trainers Groups.Lastly, some of the comments about CSR and ESR engagement are revealing. We attempt to calibrate on our own annual trainers' conference but I think this is brought out as an extremely important point. Methods to enable this as an ongoing formative process (possibly peer review) would be worth looking at. Local Trainers groups can help but may not provide enough rigor in calibration.

Reviewer Expertise:

NA

No decision status is available

# Reviewer report: Reviewer response for version 1

**DOI:** 10.21956/mep.19642.r29272

**Published:** 2018-08-17

**Authors:** Trevor Gibbs

**Affiliations:** 1 AMEE

This review has been migrated. The reviewer awarded 4 stars out of 5 Despite the wide acceptance of portfolios and e-portfolios, there still seems to be many questions asked of their value and effect. This paper digs into the e-portfolio story and provides some interesting findings.I thought that the paper was well structured, very easy to read and with a very useful introductory section with very useful references. I am not sure as to why the authors avoided a focus group approach, given that in my views the focus groups allow for much more open and informative discussion, especially if run over time. Also, the low numbers of participants and the fact that they appear to come from the same training group may actually skew the results , dependent upon how the trainees were "taught" about e-portfolios and their uses. I wonder how many of the participants had used e-portfolios in their undergraduate years and how much "training / teaching" they had experienced during previous teaching activities.I would very much agree with the author's final bullet point, which suggests to me that for many situations the personal element of portfolios- exploring the inners of the student - is often not explored, with the faculty concentrating on both the summative element and the clinical portion.Despite my reservations, I would recommend this paper to all those involved in developing portfolios at whatever level and in whatever form.

Reviewer Expertise:

NA

No decision status is available
